# Infection prevention practices and its associated factors among hospital workers in a national medical center designated for COVID-19 in Tokyo, Japan

**DOI:** 10.1371/journal.pone.0272856

**Published:** 2022-08-11

**Authors:** Rachana Manandhar Shrestha, Yosuke Inoue, Ami Fukunaga, Dong Van Hoang, Shohei Yamamoto, Takako Miki, Maki Konishi, Norio Ohmagari, Tetsuya Mizoue

**Affiliations:** 1 Department of Epidemiology and Prevention, Center for Clinical Sciences, National Center for Global Health and Medicine, Shinjuku-ku, Tokyo, Japan; 2 Disease Control and Prevention Center, National Center for Global Health and Medicine, Shinjuku-ku, Tokyo, Japan; National Center for Geriatrics and Gerontology, JAPAN

## Abstract

**Background:**

While healthcare workers (HCWs) are at risk of occupational exposure to SARS-CoV-2 infection, the virus transmission involving them might be exceeding in the non-occupational settings. This study examined the extent of adherence to infection prevention practices (IPPs) against COVID-19 in their daily life and its associated factors among staff members in a national medical center designated for COVID-19 treatment in Tokyo, Japan.

**Methods:**

This cross-sectional study was conducted in July 2020 among 1,228 staff of National Center for Global Health and Medicine (NCGM). We asked participants about their adherence on six IPPs recommended by the WHO in their daily lives, which included wearing masks, maintaining hand and respiratory hygiene, avoiding 3Cs and social distancing. We defined 100% adherence (6 points) to IPPs as good adherence and run logistic regression model to estimate odds ratios (ORs) and 95% confidence intervals (CIs) of IPPs.

**Results:**

Nearly 100% of NCGM staff members adhered to four out of six IPPs assessed in this study: washing or sanitizing hands (99.6%), good cough etiquette (99.6%), wearing mask (98.9%), and avoiding 3Cs (98.3%). Doctors (AOR = 2.18, CI: 1.36–3.49) and female staff members (AOR = 1.95, CI: 1.36–3.49) were more likely to adhere to IPPs compared with non-clinical staffs and male counterparts. Good adherence to IPPs tended to increase with older age, with highest adherence among those who were 50 years or above (AOR = 2.53, CI: 1.49–4.29).

**Conclusion:**

This study revealed that the IPPs among NCGM staff was remarkably good. Older and female staff members, and doctors showed a higher adhere to IPPs compared with their counterparts. Additional effort to improve adherence to IPPs among the younger and male staff members could contribute to reduce infection risk in their daily life, which can eventually prevent nosocomial infection.

## Background

While healthcare workers (HCWs) are the key members in combating Coronavirus Disease 2019 (COVID-19) pandemic, they are at a substantial risk of occupational exposure to severe acute respiratory syndrome coronavirus 2 (SARS-CoV-2) [1]. It was estimated that over 10–20% of the global infections are accounted for by them [2, 3]. To protect HCWs from risk of occupational exposure to the virus, the World Health Organization (WHO) has recommended personal protective equipment (PPE) use, hand hygiene practices and universal masking policies in healthcare facilities [2].

Recent studies have reported that virus transmission involving HCWs in non-occupational settings might be exceeding than in occupational settings [4–6].This warrants the need of additional effort to prevent exposures to the virus in their daily life. In order to protect HCWs from the risk of non-occupational exposures to the virus, several infection prevention practices (IPPs) recommended by the WHO such as social distancing, avoiding 3Cs (crowded places, closed spaces and close-contact) may be key elements [2, 7].

Evidence is limited on adherence to IPPs against SARS-CoV-2 among the HCWs in their daily life globally [8–10]. In addition, available evidence did not consider some of the important practices such as avoiding 3Cs, maintaining good cough etiquette and avoid touching facial areas recommended by the WHO [7].

Therefore, this study aimed to examine the proportions of staff’s good adherence to IPPs recommended by the WHO against COVID-19 and its associated factors among staff members at National Center for Global Health and Medicine (NCGM), one of the major COVID-19 designated medical centers in Tokyo.

## Methods

### Study setting

NCGM is one the six national centers for advanced and specialized medicine and is a major institution in infectious diseases control in Japan. It has been taking a lead role in the treatment of patients with SARS-CoV-2 and COVID-19-related research since the beginning of the pandemic [11]. The center has been taking comprehensive protective measures to control the infection spread in the hospital and protect its staff members from the exposure to the virus in the occupational as well as non-occupational settings [12]. These measures included sufficient provision of PPE for HCWs at higher risk of exposure to infection, universal masking policy, and intensive and frequent hand washing/ sanitizing practice. It has also introduced some practices such as partitioning dining table with acrylic board, maintaining social distance and strictly restricting communication while dining at the cafeteria, and advisory e-mail message about IPPs sent to staff every Friday to increase adherence during the weekend.

### Study design and participants

This study is cross-sectional in design. We collected data in July 2020 (after the first wave of epidemic in Japan) during the first round of the Clinical Epidemiology Study on SARS-CoV-2 Antibody, which is an ongoing survey conducted to estimate the seroprevalence of SARS-COV-2 antibodies among staff members at NCGM.

**Fig 1:**
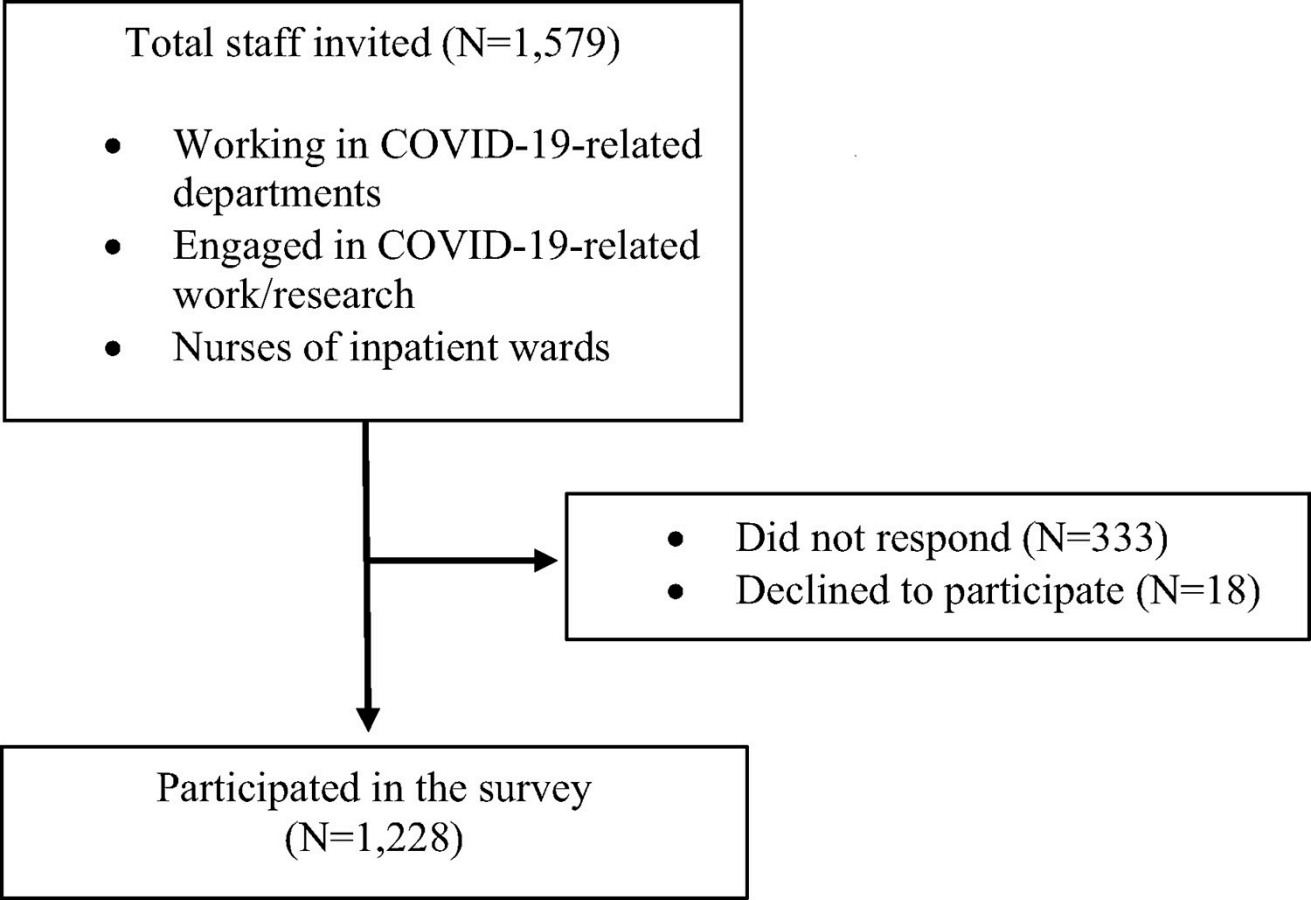
Flow diagram of study participants.

In collaboration with human resources division, we invited staff members who worked in the COVID-19-related departments, who engaged in COVID-19-related work or research, and nurses in the inpatient wards. Of 1,579 eligible staff members who were sent an invitation to the study, 333 did not respond and 18 declined to participate, leaving 1,228 (77.8%) for the present study. We have no data comparing background characteristics between participants and non-participants because we are not allowed to use the contact information for non-participants. Fig 1 shows the flow diagram of the study participants. We used an electronic survey system, where participants can return their answer only after replying to all the questions. The study was approved by the NCGM Ethics Committee (approval number: NCGM-G-003598-00) and informed consent was obtained from all the participants.

### Measures

#### Infection prevention practices

We asked participants how often they practiced the following six IPPs in past one month: (1) avoiding 3Cs (crowded places, close-contact settings and confined and enclosed spaces); (2) social distancing (2 meters; if not possible, 1 meter); (3) wearing a mask when talking or when you are outdoors; (4) practicing good cough etiquette; (5) trying not to touch eyes, nose, and mouth; and (6) washing or sanitizing hands. Participants’ responses were in a 4-point Likert scale: “always”, “often”, “seldom”, and “not at all”, with the first two response options regarded as good adherence to the practice (i.e., a score of 1). We obtained the total IPPs score (ranging from 0 to 6) and defined 100% adherence (6points) to IPPs as good adherence [8].

#### Exposures

Information was collected on participants’ age which was categorized in four groups (i.e., <30, 30-<40, 40-<50, or ≥50 years old), sex (male or female) and living arrangement (living alone to living with 2, 3, 4, or ≥5 people). We then recategorized living arrangement into two groups as “living alone” and “living with others”.

We obtained information on participants’ job categories (doctors, nurses, allied HCWs, clerical and administrative staff, researchers, workers at the Bureau of International Cooperation, medical social worker, medical professional in clinical trial development office and other workers such as car driver, laundry cleaner etc.) and the departments from the labor management office. For the job categories, we merged clerical and administrative staff, researchers, workers at the Bureau of International Cooperation, medical social worker, medical professional in clinical trial development office and other workers into the category “non-clinical staff” because of small sample size in each of those groups. For departments, we created two categories from nine categories based on their possible contact with patients with COVID-19 as “COVID-related” (COVID unit and emergency unit), and “non-COVID related” departments.

We assessed participants’ level of exposure to the virus using the following questions: “Have you ever engaged in the COVID-19-related work?” and “Did you engage in any work in which you were heavily exposed to the SARS-CoV-2 infection?”. We then categorized the participants into three groups according to potential risk of infection: low (i.e., who did not engage in COVID-19-related work), moderate (i.e., engaged in COVID-19-related work, but without heavy exposure to the virus), and high (i.e., engaged in COVID-19-related work with heavy exposure to the virus).

We assessed co-morbid condition in participants as “yes” if the participants had any one of the following chronic conditions such as diabetes, hypertension, chronic obstructive pulmonary disease (COPD), heart disease, cerebrovascular disease, cancer, and other chronic diseases, which could increase severity of COVID-19 infections.

### Data analysis

We used descriptive statistics to examine participants’ adherence to each item of IPPs. We then conducted bivariate and multivariate logistic regression analyses to estimate odds ratios (ORs) and 95% confidence intervals (CIs) of IPPs. In the multivariable logistic regression, we adjusted for age, sex, living arrangement, job categories, departments, degree of possible exposure to virus and co-morbidities. We reported adjusted odds ratio (AOR) and 95% confidence intervals, and the level of significance was set at p*<* 0.05 (two-tailed). We used Stata version 15 (College Station, TX, USA) for all statistical analyses.

## Results

In this study, almost 37.9% of the participants were below 30 years old and 71.2% were female (Table 1). Regarding occupational background, 48.9% were nurses, 10.4% worked in COVID-related departments, and 27.8% of them had high possibility to exposure to the virus. More than half of the participants (54.9%) were living together with one or more people.

**Table 1:** Characteristics of study participants at National Center for Global Medicine and Health in Tokyo, Japan (n = 1,228).

Variables	
**Total, n (%)**	1,228
**Age in years, n (%)**	
< 30	465 (37.9)
30-<40	335 (27.3)
40-<50	257 (20.9)
≥50	171 (13.9)
**Sex, n (%)**	
Male	353 (28.8)
Female	875 (71.2)
**Living arrangement, n (%)**	
Living alone	554 (45.1)
Living with others	674 (54.9)
**Job categories, n (%)**	
Non-clinical staff*	221 (18.0)
Doctors	237 (19.3)
Nurses	601 (48.9)
Allied healthcare professionals	169 (13.8)
**Departments, n (%)**	
COVID-19-related	128 (10.4)
Non-COVID-19-related	1,100 (89.6)
**Degree of possible exposure to SARS-CoV-2, n (%)**	
Low	408 (33.2)
Moderate	478 (38.9)
High	342 (27.8)
**Co-morbidities, n (%)** ^#^	
No	1, 021 (83.1)
Yes	207 (16.9)

*Non-clinical staff = Clerical and administrative staff, researchers, workers at the Bureau of International Cooperation, Medical Social Worker, Medical professional in clinical trial development office, car driver, laundry cleaner

^#^ Co-morbidities = diabetes, hypertension, COPD, heart disease, CVD, cancer, and other chronic conditions

Table 2 depicts the proportions of participants with good adherence to each item of IPPs, shown by their job categories, departments, and degree of possible exposure to virus. Almost everybody adhered to the recommended practices in relation to avoiding 3Cs (98.3%), wearing mask (98.9%), good cough etiquette (99.6%) and washing or sanitizing hands (99.6%). The proportions of those with good adherence were relatively lower for social distancing (87.9%), and avoiding touching eyes, nose, and mouth (86.3%).

**Table 2:** Adherence to infection prevention practices among study participants at National Center for Global Medicine and Health in Tokyo, Japan (n = 1,228).

	Infection Prevention Practices n (%)					
	Avoid 3Cs	Social distancing	Wear mask	Good cough etiquette	Not to touch eyes, nose, and mouth	Wash or sanitize hands
**All participants, n (%)**	1,207 (98.3)	1,076 (87.6)	1,214 (98.9)	1,225 (99.8)	1,060 (86.3)	1,223 (99.6)
**Job categories, n (%)**						
Non-clinical staff*	214 (96.8)	189 (85.5)	216 (97.7)	221 (100.0)	185 (83.7)	220 (99.5)
Doctors	235 (99.2)	215 (90.7)	236 (99.6)	237 (100.0)	209 (88.2)	234 (98.7)
Nurses	593 (98.7)	524 (87.2)	593 (98.7)	600 (99.8)	522 (86.9)	601 (100.0)
Allied health-care professionals	165 (97.6)	148 (87.6)	169 (100.0)	167 (98.8)	144 (85.2)	168 (99.4)
**Departments, n (%)**						
COVID-19-related	128 (100.0)	122 (95.3)	126 (98.4)	128 (100.0)	101 (78.9)	127 (99.2)
Non-COVID-19-related	1,079 (98.9)	954 (86.7)	1,088 (96.3)	1,097 (99.7)	959 (87.2)	1,096 (99.6)
**Degree of possible exposure to SARS-CoV-2, n (%)**						
Low	403 (98.8)	345 (84.6)	400 (98.0)	408 (100.0)	352 (86.3)	408 (100.0)
Moderate	469 (98.1)	421 (88.1)	476 (99.6)	478 (100.0)	414 (86.6)	474 (99.2)
High	335 (97.9)	310 (90.6)	338 (98.8)	339 (99.1)	294 (86.0)	341 (99.7)

*Non-clinical staff = Clerical and administrative staff, researchers, workers at the Bureau of International Cooperation, Medical Social Worker, Medical professional in clinical trial development office, car driver, laundry cleaner

Table 3 shows the results of logistic regression analysis investigating the factors associated with good adherence to IPPs. Doctors (AOR = 2.18, CI: 1.36–3.49) and female staff members (AOR = 1.95, CI: 1.36–3.49) were more likely to adhere to IPPs compared with non-clinical staffs and male counterparts. Good adherence to IPPs tended to increase with older age, with highest adherence among those who were 50 years or above (AOR = 2.53, CI: 1.49–4.29). We did not find any evidence of significant association in relation to staffs’ departments, and the degree of possible exposure to virus.

**Table 3:** Factors associated with good adherence to infection prevention practices among study participants at National Center for Global Medicine and Health in Tokyo, Japan (n = 1,228).

Variables	Infection prevention practices Good adherence, n (%) ^+^	COR (95% CI)	AOR (95% CI) ^$^
**Age in years**			
< 30	336 (72.2)	ref	ref
30-<40	256 (76.4)	1.24 (0.89–1.71)	1.33 (0.94–1.89)
40-<50	205 (79.8)	**1.51 (1.04–2.18)**	**1.63 (1.09–2.46)**
≥50	144 (84.2)	**2.04 (1.29–3.23)**	**2.53 (1.49–4.29)**
**Sex**			
Male	255 (72.2)	ref	ref
Female	686 (78.4)	**1.39 (1.05–1.85)**	**1.95 (1.36–2.80)**
**Living arrangement**			
Living alone	413 (74.6)	ref	ref
Living with others	528 (78.3)	1.23 (0.94–1.60)	1.09 (0.80–1.48)
**Job categories**			
Non-clinical staff	90 (70.3)	ref	ref
Doctors	192 (81.0)	**1.62 (1.04–2.52)**	**2.18 (1.36–3.49)**
Nurses	463 (77.0)	1.27 (0.90–1.82)	1.35 (0.89–2.05)
Allied health-care professionals	126 (74.6)	1.11 (0.70–1.76)	1.37 (0.84–2.25)
**Departments**			
Non-COVID-19-related	844 (76.7)	ref	ref
COVID-19-related	97 (75.8)	0.94 (0.61–1.45)	0.88 (0.55–1.40)
**Degree of possible exposure to SARS-CoV-2**		
Low	304 (74.5)	ref	ref
Moderate	370 (77.4)	1.17 (0.86–1.59)	1.27 (0.92–1.75)
High	267 (78.1)	1.21 (0.86–1.71)	1.38 (0.94–2.01)
**Co-morbidities** ^**#**^			
No	775 (75.9)	ref	ref
Yes	166 (80.2)	1.28 (0.88–1.86)	1.15 (0.83–1.83)

^**$**^Adjusted for age (<30, 30-<40, 40-<50, or ≥50 years old), sex (male or female), living arrangement (alone or with others), job categories (non-clinical staff, doctors, nurses, and allied health-care professionals), departments (COVID-19 related or non-COVID-19-related), degree of possible exposure to SARS-CoV-2 (low, moderate or high), and co-morbidities (yes or no), CI = Confidence Interval, COR = Crude Odds Ratio, AOR = Adjusted Odds Ratio

*Non-clinical staff = Clerical and administrative staff, researchers, workers at the Bureau of International Cooperation, Medical Social Worker, Medical professional in clinical trial development office, car driver, laundry cleaner

^#^ Co- morbidities = diabetes, hypertension, COPD, heart disease, CVD, cancer, and other chronic conditions; ^**+**^Adherence to the six-infection prevention practices (i.e., avoiding 3Cs, social distancing, wearing mask, good cough etiquette, avoid touching eyes, nose and mouth, and wash or sanitize hands) was defined as good adherence.

## Discussion

In this study, we found that the proportions of staffs with good adherence to each IPPs was quite high at the NCGM, which is a national medical center designated for the COVID-19 treatment in Tokyo, Japan. Female and older staff members and doctors were more likely to adhere to IPPs.

Nearly 100% of NCGM staff adhered to four out of six IPPs assessed in this study: washing or sanitizing hands (99.6%), good cough etiquette (99.6%), wearing mask (98.9%), and avoiding 3Cs (98.3%) in their daily lives. Such high adherence is reasonable given the significant role of NCGM against this infection. NCGM staffs have been involved in various COVID-19-related mission since the early phase of the epidemic, and they were highly aware of and well prepared for the infection prevention. The infection control department of the center have send an e-mail message regarding infection prevention to the staffs on weekly basis [13], which might have strengthen their awareness of IPPs. Meanwhile, we previously reported a low seroprevalence of SARS-CoV-2 (0.16%) among NCGM staffs as of July 2020 [13]. This finding may be ascribed, at least in part, to their good adherence to IPPs as well as the comprehensive measures taken by the center to protect them.

The proportion of the participants adhering to avoid touching eyes, nose and mouth was lower than those for the four IPPs mentioned above, yet 86.3% of them reported adherence to it, which was still quite high. A previous study showed decrease in face-touching behavior during vs. before COVID-19 pandemic in Japan [14]. The universal masking campaign [13] might have influenced face-touching behavior during the pandemic.

Social distancing was also relatively low (87.6%) among the study participants. One possible interpretation is that social distancing can be difficult in the public areas such as trains, cafeterias, and restaurants, where we cannot avoid or control other people’s physical proximity. A study conducted among Lebanese physicians, also reported maintaining social distance was practiced the least (55.3%) compared to other IPPs such as washing hands (96.6%), wearing masks (89.5%), gloves (65.3%) and PPE gowns (75.5%) [8].

In the analysis by job categories, we found that doctors were more likely to have good adherence to IPPs compared with non-clinical staffs. Our finding is similar to that of a previous study in Ghana [15], which showed better compliance to infection preventive practices among clinical staffs compared with non-clinical staffs during the COVID-19 pandemic. As the clinical staffs are well trained to strictly adhere to IPPs in the hospital, they might be more likely to extend their practice to daily life than other staffs. We hypothesized that staff members working in COVID-19-related departments, those with higher exposure to SARS-CoV-2 virus, and those with comorbidity could more likely to adhere to IPPs based on previous studies by Abbas et al. [8] and Ejaz et al. [16], respectively. Contrary to our expectation, we did not find significant difference in IPPs in relation to their affiliation, occupational infection risk, and comorbidity, suggesting that these factors may not independently influence daily life preventive practices among HCWs with high level of adherence to IPPs.

Our findings of better adherence to IPPs among female and older staff members compared with male and younger counterparts, respectively, were consistent with those of previous studies. A study in India reported that older and female HCWs were more likely to adhere to IPPs against COVID-19 such as hand hygiene, wearing mask and social distancing [10]. In a population study in Japan during COVID-19 pandemic, female and older adults practiced personal protective measures more than male and younger adults did [17]. In a study in Ethiopia conducted before COVID-19 pandemic, older HCWs were more likely to adhere to IPPs than younger counterparts [18]. Male staff members were less likely to adhere to IPPs compared with female counterparts could be due to gender difference in COVID-19-related belief. For example, study by Galasso et al. conducted in eight countries reported that women were more likely to perceive COVID-19 as a serious health problem than men and showed higher compliance to IPPs [19]. Lower adherence to IPPs among younger staff members could be ascribed to their insufficient knowledge and experience, and lower level of perceived threat [8, 9, 20]. Our findings suggest that male and younger staffs might be at greater risk of infection compared with female and older counterparts. Awareness messages targeting these groups may encourage them to adhere to IPPs more.

The findings of this study should be interpreted with a few study limitations. First, we cannot ignore the possibility of selection bias. For example, the staff members with good adherence to IPPs might be more likely to participate in the survey, leading to an overestimation of IPPs. Second, this study was conducted in one of the national medical centers designated to treat patients with COVID-19 and convenient sampling method was applied to recruit study participants, therefore, the findings may not be generalized to other settings. Third, the data in this study was self-reported by the participants, which might have led to recall bias. Fourth, the possibility of overestimation of IPPs taken by the staff during the pandemic cannot be ignored because they might have presented themselves in a manner desirable during the pandemic, leading to social desirability bias. Lastly, regarding living arrangement, our study lacked information about the space and the way people were living which can affect infection transmission and may influence IPPs.

## Conclusion

This study revealed that the IPPs among NCGM staff was remarkably good. Older and female staff members, and doctors showed a higher adherence to IPPs compared with their counterparts. Additional effort to improve adherence to IPPs among the younger and male staff members could contribute to reduce infection risk in their daily life, which can eventually prevent nosocomial infection.
